# Strand-specific RNA-Seq transcriptome analysis of genotypes with and without low-phosphorus tolerance provides novel insights into phosphorus-use efficiency in maize

**DOI:** 10.1186/s12870-016-0903-4

**Published:** 2016-10-10

**Authors:** Qingguo Du, Kai Wang, Cheng Xu, Cheng Zou, Chuanxiao Xie, Yunbi Xu, Wen-Xue Li

**Affiliations:** National Key Facility for Crop Gene Resources and Genetic Improvement, Institute of Crop Science, Chinese Academy of Agricultural Sciences, Beijing, 100081 China

**Keywords:** Maize, Genotype, Phosphorus, Strand-specific RNA-Seq, Differential gene expression, ROS

## Abstract

**Background:**

Phosphorus (P) stress is a global problem in maize production. Although macro/microarray technologies have greatly increased our general knowledge of maize responses to P stress, a greater understanding of the diversity of responses in maize genotypes is still needed.

**Results:**

In this study, we first evaluated the tolerance to low P of 560 accessions under field conditions, and selected the low P-tolerant line CCM454 and the low P-sensitive line 31778 for further research. We then generated 24 strand-specific RNA libraries from shoots and roots of CCM454 and 31778 that had been subjected to P stress for 2 and 8 days. The P deficiency-responsive genes common to CCM454 and 31778 were involved in various metabolic processes, including acid phosphatase (APase) activity. Determination of root-secretory APase activities showed that the induction of APase by P stress occurred much earlier in CCM454 than that in 31778. Gene Ontology analysis of differentially expressed genes (DEGs) and CAT/POD activities between CCM454 and 31778 under P-sufficient and -deficient conditions demonstrated that CCM454 has a greater ability to eliminate reactive oxygen species (ROS) than 31778. In addition, 16 miRNAs in roots and 12 miRNAs in shoots, including miRNA399s, were identified as DEGs between CCM454 and 31778.

**Conclusions:**

The results indicate that the tolerance to low P of CCM454 is mainly due to the rapid responsiveness to P stress and efficient elimination of ROS. Our findings increase the understanding of the molecular events involved in the diversity of responses to P stress among maize accessions.

**Electronic supplementary material:**

The online version of this article (doi:10.1186/s12870-016-0903-4) contains supplementary material, which is available to authorized users.

## Background

Phosphorus (P) is essential for the normal growth and development of plants because it is required for the regulation of energy metabolism, enzymatic reactions and signal transduction processes [1]. Plants acquire P in the form of orthophosphate. Though P is abundant in soil, it often forms insoluble complexes, particularly with aluminum and iron under acidic conditions and with calcium under alkaline conditions [2]. In addition to its slow diffusion, the low availability of P is a major environmental constraint for crop productivity worldwide [2, 3]. To obtain high yields, farmers have often added excessive quantities of P fertilizer [4], which mainly originate from nonrenewable rock phosphate. These large inputs of external P have led to a decrease in P-use efficiency. P-use efficiency is often less than 20 % and the remaining P becomes immobile in the soil or pollutes water bodies [5, 6]. One effective way to overcome these problems is to understand the genetic mechanisms of low-P tolerance in plants and to breed crop cultivars with enhanced P-use efficiency.

To reduce the adverse effects of P stress, plants have evolved several strategies, including the re-programming of root morphology to increase exploratory and absorptive capacity [7], the increased production and exudation of organic acid and phosphatases [3], the establishment of symbiotic relationships with arbuscular mycorrhizal fungi [8], and the bypassing of the metabolic steps that require ATP [9]. These adaptations in response to variable P availability are at least partially dependent on changes in gene expression. Some key regulators of P homeostasis, which have mainly been characterized from *Arabidopsis* and rice, include the MYB transcription factor PHR1, which functions as the central regulator of downstream genes [10]; members of WRKY [11–15] and PHO families [16, 17]; the miRNAs miRNA399 and miRNA827 [18, 19]; E3 ligase NLA and SIZ1 [19, 20]; and IPS1/At4 [21, 22]. In contrast, only a bHLH transcription factor, *ZmPTF1*, has been demonstrated to increase low-P tolerance in maize; it does so by regulating carbon metabolism and root growth [23].

Maize ranks first in total production among major staple cereals and is not only a worldwide food and feed crop but also is an important raw material for energy production and many other industrial applications [24]. Maize yield, however, is frequently threatened by various abiotic stresses, including low-P stress, especially in the acidic and alkaline soils of tropical and subtropical regions [25]. Macro/microarray technologies have greatly increased our understanding of the molecular mechanisms regulating P homeostasis in plants [26–28]. By using an oligonucleotide microarray platform, Calderon-Vazquez et al. detected a total 1179 P-stress responsive genes (normal P vs. low P) in the roots of a low P-tolerant maize genotype; among these genes, at least 33 % lack an orthologue in the *Arabidopsis* genome [25], suggesting that some P responsive pathways are unique in maize [29].

The probes used in arrays for maize gene studies, however, were designed based on the past knowledge of maize gene annotation. As an alternative to macro/microarray technologies, high-throughput sequencing can be used to study the molecular basis of P-stress tolerance in maize. Compared to macro/microarray technologies, probe-free high-throughput sequencing is more sensitive and more effective at identifying nuclear transcripts, DNA repair, and chromatin modifications [30]. Traditional RNA-Seq could not distinguish the sequencing data from the first- and second-strand cDNA because of the lack of RNA polarity information. Strand-specific RNA-Seq overcomes this limitation and provides more accurate information than traditional RNA-Seq for digital gene expression analysis and genome annotation [31].

Although transcriptomics based on microarray platforms have greatly increased our general understanding of maize responses to P stress, a more detailed understanding of the diversity of responses in maize genotypes is needed [29]. In the present research, we evaluated 560 maize accessions for low-P tolerance under field conditions in 2014 and 2015, and we selected two lines, 31778 and CCM454, that differed in their tolerance to low P for further research. Based on the physiological indices tested, we used strand-specific RNA-Seq transcriptome analyses of leaves and roots of low P-tolerant and -sensitive maize inbred lines to explain the molecular mechanisms of genotypic diversity in maize in response to P stress. This research increases the understanding of the genetic variations and molecular basis of low-P tolerance in maize.

## Results

### Selection of genotypes with and without low-P tolerance in field and hydroponic experiments

In the field experiment, 15 accessions with low-P tolerance and 15 with low-P sensitivity were identified. The accessions with low-P tolerance were Huang4283, Te70, Q1261, Dan598, 888–9, Xi14, 7537–1, CCM26, CCM481, CCM454, Mo17, Si273, Dan599, CCM1143, and Hai9-21. The accessions with low-P sensitivity were 5022, Zheng30, Si387, Liao540, 706Fu, Qi205, Ji853, 31778, FR19, 1538, B73, CA091, Liao5114, CCM111, and Ji419. Consistent with previous reports [32], inbred line Mo17 was found to be low-P tolerant, and inbred line B73 was found to be low-P sensitive.

**Fig. 1:**
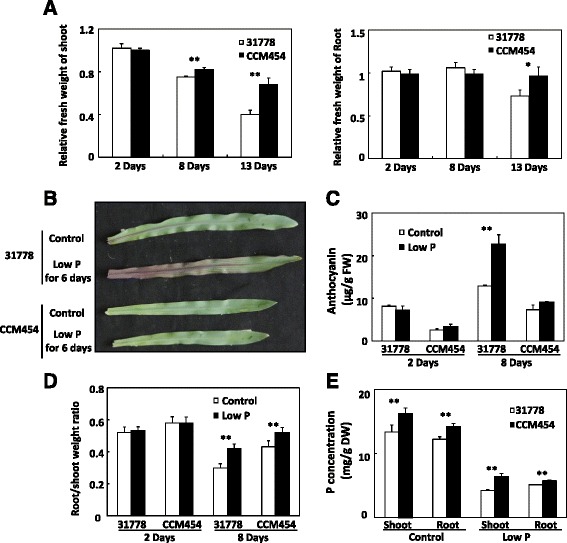
Phenotypic and physiological responses of maize inbred lines 31778 and CCM454 to P stress. 31778 and CCM454 seedlings were grown in a hydroponic solution containing 150 μM or 5 μM Pi for the indicated durations. **a** Relative fresh weight of shoot and root (−P vs. +P); **b** Photographs of representative plants; **c** Anthocyanin content of shoots; **d** Root/shoot weight ratio; and **e** P concentrations in shoot and root of inbred lines 31778 and CCM454. For A, C, D, and E, values are means ± SE (*n* = 5). *Asterisks* indicate significant differences as determined by t tests (** *P* < 0.01, * *P* < 0.05)

Inbred lines CCM454 (low-P tolerant) and 31778 (low-P sensitive) were selected for further research because neighbour joining tree analysis indicated that these lines are closely related. We first investigated their responses to P stress in hydroponic solutions containing sufficient (150 μM) or limiting (5 μM) P. At the onset of treatment, relative fresh weight of shoot and root, anthocyanin levels and root/shoot weight ratio of both CCM454 and 31778 were similar between P-sufficient and -deficient conditions (Fig. 1). When plants were transferred to the P-deficient medium for 8 days, the shoot fresh weight decreased by 25 % for 31778 and by 18 % for CCM454 (Fig. 1a). This difference between 31778 and CCM454 increased when the P-deficient treatment was extended to 13 days (60 % vs. 32 %) (Fig. 1a). A phenotypic difference between inbred lines CCM454 and 31778 was evident at 6 days after P-deficient treatment, when an accumulation of the purple flavonoid pigment anthocyanin in older leaves was observed in 31778 but not in CCM454 (Fig. 1b). The anthocyanin levels in 31778 after 8 days of P stress was ~23 μg/g fresh weight (Fig. 1c), which was about 3 times higher than the level in CCM454. Compared with the C allocation under the P-sufficient condition, a higher proportion of C was allocated to roots after P deficiency for 8 days, especially for the low-P tolerant CCM454 (Fig. 1d). The root-to-shoot weight ratios were much higher for CCM454 than for 31778 regardless of P treatment for hydroponically grown 8-day plants (Fig. 1d). P deficiency led to a significantly decrease in P concentration in the shoots and roots of both 31778 and CCM454 (Fig. 1e). However, the total P concentration in the shoots of 31778 was 4.2 mg/g DW after P deficiency for 8 days, which was ~51 % lower than that of the CCM454 (Fig. 1e). Similar results were obtained for roots (Fig. 1e). These results indicate that CCM454 is more tolerant to low-P stress than 31778 even under hydroponic conditions.

### RNA-Seq transcriptome of genotypes with and without low-P tolerance

To identify molecular events involved in low-P tolerance, a total of 24 RNA libraries from shoots and roots of both inbred lines CCM454 and 31778 were generated. As noted earlier, the plant samples were obtained from hydroponically grown seedlings that had been provided with sufficient P for 2 days or low P for 2 or 8 days. Each sample was represented by two biological replicates, and the libraries were sequenced by Illumina high-throughput sequencing technology. These RNA libraries yielded a total of more than 2.1 billion reads after adaptor trimming, and approximately 77 % of the clean reads could be perfectly mapped to maize B73 RefGen_V3.27 (ftp://ftp.ensemblgenomes.org/pub/plants/release-27/fasta/zea_mays) (Additional file 1). Sequences that could not be mapped to the maize genome were discarded, and only those perfectly mapped were analyzed further. The transcripts were then classified into exon, intron, and intergenic region (Additional file 1).

**Fig. 2:**
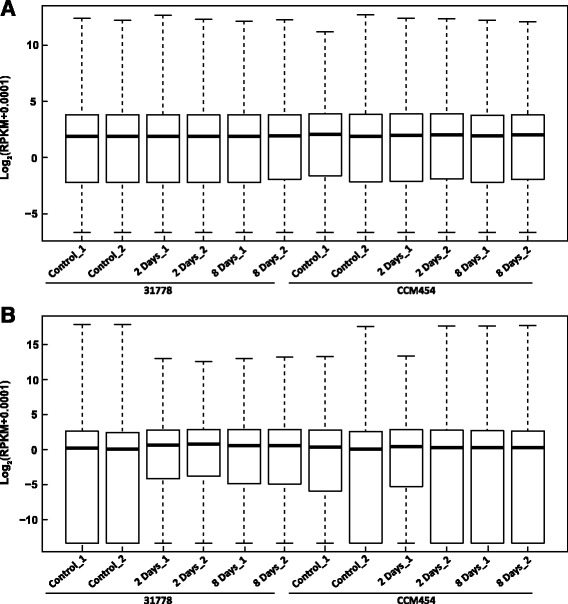
Boxplot of the log_2_(RPKM + 0.0001) expression values of roots **a** and shoots **b** of maize inbred lines 31778 and CCM454

The abundance of each gene was determined by reads per kilobase million mapped reads (RPKM) [33]. The median values of Log_2_(RPKM + 0.0001) among different libraries used for differential expression assessment were comparable (Fig. 2). We also calculated the correlation of the two biological replicates for each treatment to investigate the variability between the replicates. The pearson’s correlations (R value) of almost all comparisons exceeded 90 % (Additional file 2), indicating a high correlation between biological replicates.

We further confirmed the RNA-Seq transcriptome by real-time RT-PCR. In agreement with our RNA-Seq data, the real-time RT-PCR assay showed that P stress strongly up-regulated the expression of GRMZM2G475536, GRMZM2G152447, GRMZM2G112377, GRMZM2G436295, GRMZM2G423898, GRMZM2G333183, GRMZM2G135839, GRMZM2G477503, and *MIR399j* but down-regulated the expression of GRMZM2G170742, GRMZM2G001205, GRMZM2G011006, GRMZM2G046952, AC198414.2_FG001, GRMZM2G428216, GRMZM5G856297, GRMZM2G124540, and *MIR169c* (Additional file 3). These results further indicated that the sequencing data were reliable.

### P deficiency-regulated genes in genotypes with and without P tolerance

**Fig. 3:**
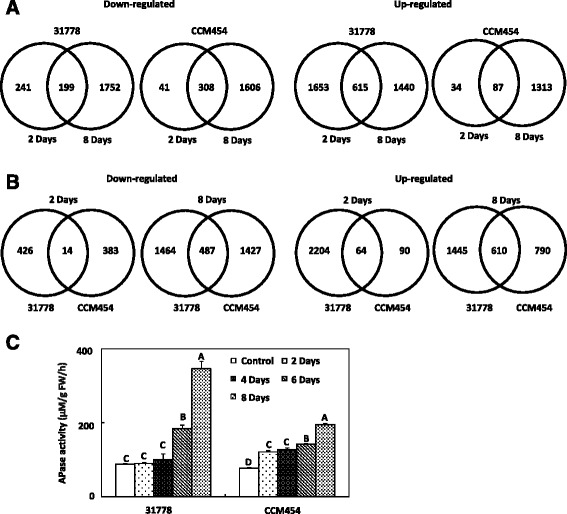
Overview of P stress-responsive genes and of root-secretory acid phosphatase activity in maize inbred lines 31778 and CCM454. **a** Venn diagram illustrating P stress-responsive genes in 31778 and CCM454. **b** Venn diagram illustrating common or differentially expressed genes between the two lines in response to P stress. **c** Activities of root-secretory acid phosphatase for root segments of 31778 and CCM454 grown under P-sufficient and P-deficient conditions. Values are means and standard errors (*n* = 5). LSD test was used to test differences between treatments. Means with the same letter were not significantly different at *P* < 0.01

A total of 5900 genes in the low-P sensitive 31778 and 3389 genes in the low-P tolerant CCM454 were differently expressed in response to Pi availability at one or more sampling times. Among the P deficiency-responsive genes, 3708 genes in 31778 and 1434 genes in CCM454 were up-regulated (Fig. 3a). When the inbred lines were subjected to P deficiency for 2 days, the total number of P deficiency-responsive genes was much lower in CCM454 than in 31778 (Fig. 3a), indicating that Pi-deficiency stress was greater in 31778 than in CCM454. P deficiency-responsive genes common to CCM454 and 31778 (487 were down-regulated genes and 610 were up-regulated genes) were detected mainly after plants had been transferred to Pi-deficient medium for 8 days (Fig. 3b). In contrast, only 64 up-regulated and 14 down-regulated genes were common to CCM454 and 31778 after 2 days of P deficiency (Fig. 3b).

The P-deficiency-responsive genes common to CCM454 and 31778 should mainly result from P stress and were not related to genotypic difference. Gene Ontology (GO; http://bioinfo.cau.edu.cn/agriGO/) analysis indicated that these genes were related to various metabolic processes (lipid metabolic process, organic acid metabolic process, secondary metabolic process, acid phosphatase activity, carbohydrate metabolic process, etc.), phosphate transmembrane transporter activity and Pi starvation responses as previously reported (Additional file 4) [28].

### APase activity

To confirm the GO analysis concerning acid phosphatase (APase) activity, we measured APase activity in CCM454 and 31778 roots. The root-secretory APase activities in both CCM454 and 31778 were significantly induced by P deficiency (Fig. 3c). After 2 days of P deficiency, the root-secretory APase activity in CCM454 was 121 μM/g FW/h, which was ~ 2 times greater than the activity when P was sufficient for 2 days. In contrast, the root-secretory APase activity in 31778 was similar under Pi-sufficient and Pi-deficient conditions even after 4 days of Pi-deficient treatment (Fig. 3c). Compared to activity under Pi-sufficient condition, the root-secretory APase activity after 8 days of Pi deficiency increased 4-fold in 31778 but increased only about 2.5-fold in CCM454 (Fig. 3c).

### Identification of DEGs in the low P-tolerant genotype vs. the low P-sensitive genotype under Pi-sufficient condition

**Fig. 4:**
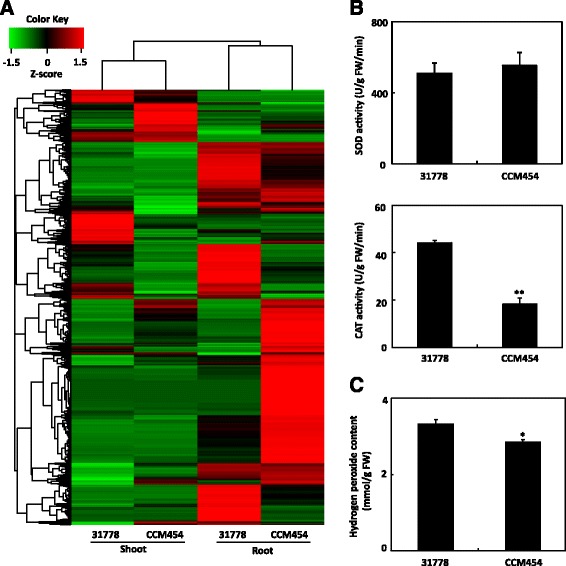
Differentially expressed genes between maize inbred lines 31778 and CCM454 under P-sufficient conditions, and SOD, CAT and H_2_O_2_ activities. **a** Heat map showing DEGs between 31778 and CCM454 under P-sufficient conditions. **b** Activities of SOD and CAT in the shoots of 31778 and CCM454 under Pi-sufficient conditions. **c** The hydrogen peroxide contents in the shoots of 31778 and CCM454. Values are means and standard errors (*n* = 4). *Asterisks* indicate significant differences between 31778 and CCM454 as determined by t tests (** *P* < 0.01, * *P* < 0.05)

Based on the criteria that the Log_2_ fold-change ratio was ≥ 1 and that the adjusted *P* value was ≤ 0.05, 3750 genes in shoots and 5230 genes in roots were identified as differentially expressed genes (DEGs) in CCM454 vs. 31778 under P-sufficient condition (Fig. 4a, Additional files 5 and 6). These DEGs were highly tissue specific, and only ~21 % were expressed in both shoots and roots (Fig. 4a). Among the DEGs (31778 vs. CCM454), 4141 genes were up-regulated and 3839 genes were down-regulated in CCM454. To determine the molecular events responsible for low-P tolerance of CCM454, we first focused on the potential functions of up-regulated genes in CCM454. The up-regulated genes in CCM454 were enriched for biological processes involved in phosphate metabolic process (GO:0006796, *P* = 1.6e^−5^, 1.5-fold enrichment), phosphorus metabolic process (GO:0006793, *P* = 1.7e^−5^, 1.5-fold enrichment), electron transport chain (GO:0022900, *P* = 2.3e^−5^, 2.4-fold enrichment), and aromatic compound catabolic process (GO:0019439, *P* = 4e^−5^, 1.6-fold enrichment).

When we analyzed the 3839 up-regulated genes in the low P-sensitive 31778, and found that these DEGs were related to inorganic anion transmembrane transporter activity (GO:0015103, *P* = 8.5e^−5^, 3.7-fold enrichment), response to stimulus (GO:0050896, *P* = 6.7e^−11^, 1.5-fold enrichment), oxidoreductase activity (GO:0016706, *P* = 0.00023, 2.4-fold enrichment) and response to abiotic stress (GO:0009628, *P* = 4.5e^−7^, 1.7-fold enrichment). These results suggested that the physiological status of the low P-sensitive 31778 might be sub-optimal even when sufficient P was provided. To test this hypothesis, the activities of two significant antioxidant enzymes, superoxide dismutase (SOD) and catalase (CAT), were measured in CCM454 and 31778 under P-sufficient conditions (Fig. 4b). SOD activity did not differ between CCM454 and 31778. However, CAT activity in 31778 was 44.2 U/g FW/min, which was about 2.5 times higher than in CCM454. The enhancement of CAT activity in 31778 might be due to an increase in H_2_O_2_ content in 31778 (Fig. 4c).

### Identification of P stress-responsive DEGs in the low P-tolerant genotype vs. the low P-sensitive genotype

**Fig. 5:**
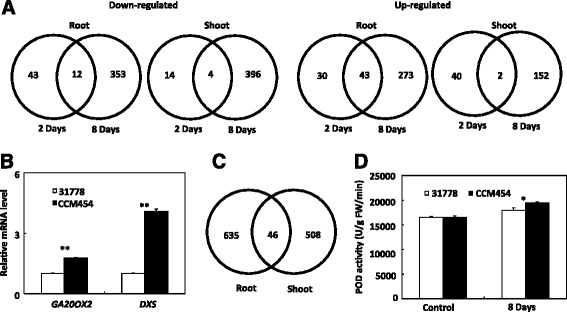
Venn diagram **a c** and real-time RT-PCR **b** analysis of differentially expressed genes between inbred lines 31778 and CCM454 under Pi-deficient conditions. Quantifications were normalized to the expression of *GAPDH*. Values are means and standard errors (*n* = 3). Activities of POD in the roots of 31778 and CCM454 grown under P-sufficient and -deficient conditions are also showed **d**. Values are means and standard errors (*n* = 4). *Asterisks* indicate significant differences between 31778 and CCM454 as determined by t tests (** *P* < 0.01, * *P* < 0.05)

To clarify the increased low-P tolerance of CCM454, we identified P stress-responsive DEGs between lines CCM454 and 31778. At the onset of Pi deficiency, the number of P stress-responsive DEGs between CCM454 and 31778 was small in both roots and shoots (Fig. 5a). However, some important genes involved in hormone synthesis, phosphate homeostasis and secondary metabolism were up-regulated in CCM454 (Additional file 7). Among these genes, *GA20OX2* (AC234528.1_FG006) is the key oxidase enzyme in the biosynthesis of gibberellin; GRMZM2G169149 encodes *ZmWRKY62*, and the members in WRKY family modulated tolerance to phosphate starvation in rice and *Arabidopsis* [11–15]; the 1-deoxy-D-xylulose 5-phosphate synthase (DXS) enzyme encoded by GRMZM2G493395 limits the 2-C-methyl-D-erythritol 4-phosphate (MEP) pathway, which is responsible for the synthesis of the common precursors to various isoprenoids including secondary messengers inositol polyphosphates (IPs) [34]. The up-regulation of *GA20OX2* and GRMZM2G493395 in CCM454 after 2 days of P stress was further verified by real-time RT-PCR assay (Fig. 5b).

A total of 681 P deficiency-responsive DEGs were found in roots and 554 in shoots after CCM454 and 31778 were transferred to the P-deficiency medium for 8 days (Fig. 5c, Additional file 8). Few of the P deficiency-responsive DEGs were common to shoots and roots (Fig. 5c). Relative to 31778, 365 P deficiency-responsive DEGs in roots and 400 P deficiency-responsive DEGs in shoots were up-regulated in CCM454 (Additional file 8). In CCM454 roots, the up-regulated P deficiency-responsive DEGs were mainly involved in response to stress (GO:0006950, *P* = 7.6e^−6^, 2.0-fold enrichment), antioxidant activity (GO:0016209, *P* = 1.3e^−6^, 9.8-fold enrichment), and peroxidase activity (GO:0004601, *P* = 1.8e^−6^, 5.9-fold enrichment). The assessment of peroxidase (POD) activities in the roots of 31778 and CCM454 confirmed that the up-regulated genes in CCM454 were concerned with antioxidant activity and peroxidase activity (Fig. 5d). In shoots, the up-regulated P deficiency-responsive DEGs were related to carbohydrate metabolic process (GO:0005975, *P* = 4.4e^−9^, 2.9-fold enrichment), carbohydrate biosynthetic process (GO:0016051, *P* = 5.2e^−8^, 5.0-fold enrichment), carboxylic acid catabolic process (GO:0046395, *P* = 2.2e^−7^, 9.7-fold enrichment), and organic acid biosynthetic process (GO:0016053, *P* = 0.0018, 2.5-fold enrichment). These metabolic processes contributed to the low-P tolerance of CCM454 were partly verified by the higher root-to-shoot weight ratios of CCM454 than that of 31778 after Pi-deficiency for 8 days (Fig. 1d). We also analyzed the 316 P deficiency-responsive DEGs in roots and 154 P deficiency-responsive DEGs in shoots that were down-regulated in CCM454. The down-regulated P deficiency-responsive DEGs were related to phosphoric ester hydrolase activity (GO:0042578, *P* = 5.5e^−7^, 4.3-fold enrichment), iron ion binding (GO:0005506, *P* = 6.3e^−7^, 2.9-fold enrichment), monooxygenase activity (GO: 0004497, *P* = 1.9e^−6^, 3.7-fold enrichment), and electron carrier activity (GO:0009055, *P* = 3e^−6^, 2.9-fold enrichment).

### P stress-responsive miRNAs

**Fig. 6:**
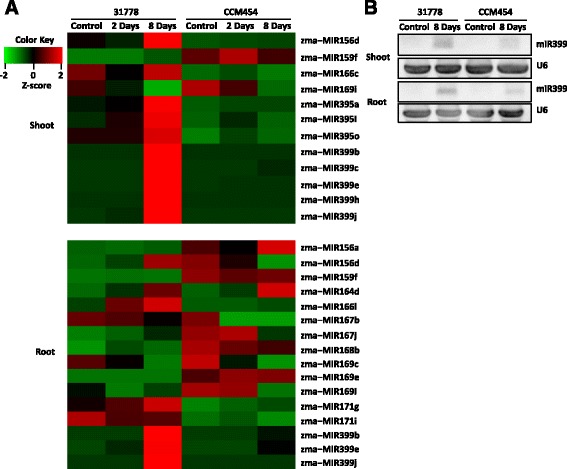
Heat map **a** and small RNA northern analysis **b** of P stress-responsive miRNAs between maize inbred lines 31778 and CCM454

Posttranscriptional gene regulation by miRNAs plays important role in plant adaptive responses to nutrient deprivation [35–38]. In the current study, 16 miRNAs belonging to nine families in roots and 12 miRNAs belonging to six families in shoots were found to be differently expressed in CCM454 vs. 33,178 under P deficiency condition (Fig. 6a). The up-regulation of miRNA399 by Pi-deficiency, which have been demonstrated to regulate Pi-deficient responses [39], was observed in the shoots and roots of the low P-sensitive inbred line 31778 only after 8 days of P deficiency (Fig. 6a). Other nutrient-responsive miRNAs, such as miRNA395 (which is involved in S-deficient responses [36]) and miRNA169 (which is related to N-starvation adaption [37]), were also differentially expressed in miRNAs between 31778 vs. CCM454. Because miRNA399 is important in phosphate homeostasis in plants, we selected miRNA399 for further validation by small RNA northern analysis. The expression of miRNA399 after 8 days of Pi deficiency was much higher in 31778 than in CCM454 (Fig. 6b), which was consistent with the sequencing data.

## Discussion

In our previous research, 826 maize germplasm including 580 tropical/subtropical accessions were evaluated for low-P tolerance in the field, and 41 low-P tolerant and low-P sensitive accessions were selected based on principal component analysis of the relative values of all traits [40]. Based on the results, we collected additional inbred lines from different ecological zones in China, CIMMYT, and USA, and evaluated their low-P tolerance. In the field screening of the current study, Mo17 was more tolerant than B73 to P stress, which agreed with a previous report [32] and which therefore indicated that our screening was reliable. This motivated us to identify the molecular events involved in the diversity of responses to P deficiency in maize genotypes. The gained information could help us develop genome-wide methods for mapping and for identifying markers [29].

Plant responses to P stress often depend on gene regulation at the posttranscriptional level. miRNA399 is induced by P stress and regulates phosphate homeostasis in *Arabidopsis*, rice, and soybean by suppressing a ubiquitin-conjugating E2 enzyme, PHO2 [34, 36, 39, 41]. In the phloem sap of rapeseed, miRNA399 abundance depends on P status [38], suggesting that miRNA399 might act as a systemic signal. This inference was further supported by a grafting experiment, which showed that a root-derived deficiency signal induces miRNA399 expression in the shoots; the induced miRNA399 is then delivered to the roots where it targets PHO2 transcripts for degradation [42]. In both shoots and roots, miRNA399 abundance was much higher in the low P-sensitive inbred line 31778 than in the low P-tolerant inbred line CCM454. In addition, the total number of P deficiency-responsive genes was also higher in 31778 than in CCM454 after P deficiency for 2 days. These results indicated that the low P-sensitive inbred line experienced greater P stress than the low P-tolerant inbred line.

In several cases, research has demonstrated that altering the expression of a transcription factor can alter resistance to P stress by activating downstream target genes. The transcription factors in question include NAC, MYB, WRYK, ERF/AP2, zinc finger proteins, CCAAT-binding transcription factor, and members of bHLH families [43–46]. Among the P stress-responsive DEGs in the low P-tolerant line vs. the low P-sensitive line in the current study, we identified 11 NACs, 11 MYBs, 10 bHLHs, 6 zinc finger proteins, 4 WRKYs, and 4 SPX domain-containing proteins. We also identified the calmodulin-binding transcription activator, bZIP transcription activator, and C2C2-GATA transcription factor as P stress-responsive DEGs in CCM454 vs. 31778. These results suggest that transcriptional regulation is important for low-P tolerance.

Under Pi-sufficient conditions, 8980 DEGs (3750 DEGs in shoots and 5230 DEGs in roots) were identified in CCM454 vs. 31778. These results indicate that the low P-tolerant CCM454 is genetically pre-adapted to P stress. This pre-adaptation could include the ability to efficiently eliminate ROS. In plants, ROS are continuously produced in chloroplasts, mitochondria, and peroxisomes as by-products of aerobic metabolism [47]. Because some ROS species are highly toxic, they must be rapidly detoxified by enzymatic and non-enzymatic mechanisms [48]. Deficiencies in N, P, K, and S can induce ROS production in *Arabidopsis* [49]. We hypothesize that the ability to eliminate ROS is greater in the low P-tolerant CCM454 than in the low P-sensitive 31778 based on the following evidence: (1) the up-regulated DEGs in 31778 under Pi-sufficient conditions were highly enriched in response to abiotic stress (GO:0009628); (2) when ROS increased after 8 days of P stress, the up-regulated DEGs in CCM454 were mainly related to antioxidant activity (GO:0016209); (3) POD activity was significantly higher in CCM454 than in 31778 regardless of P treatment.

Under P-deficient conditions, an important adaptive strategy for increasing P acquisition is the production of APases and their secretion from roots into the rhizosphere; in the rhizosphere, the APases can release P from organic sources [44, 46]. The importance of APases for P-stress resistance has been clearly demonstrated by the growth of the *Arabidopsis atpap10* loss-of-function mutant and *35S*::*PAP10* transgenic plants on a P-deficient medium [50]. Our GO analysis showed that the P deficiency-responsive genes common to CCM454 and 31778 are enriched in APase activity (GO:0003993). The root-secretory APase activity was also induced by P deficiency regardless of genotype. However, the root-secretory APase activity in the low P-tolerant CCM454 was significantly induced after 2 days of P-deficiency and remained high during P stress, whereas the root-secretory APase activity in the low P-sensitive 31778 was significantly induced only after 8 days of P deficiency. This indicated that the low P-tolerant line responded more rapidly than the low P-sensitive line to P deficiency.

P-deficiency down-regulated gibberellin response in *Arabidopsis* and white lupin [51, 52]; P itself, phytohormones, and universal secondary messengers, including Ca^2+^ and IPs, have been implicated in Pi local and systemic sensing and signaling pathways [53]. At the onset of P deficiency in the current study, genes involved in the biosynthesis and signal transduction of gibberellin were identified among P stress-responsive DEGs in CCM454 vs. 31778, further indicating that another important way in which CCM454 tolerates low P is by rapidly sensing a change in Pi levels in the plant.

## Conclusions

In summary, 15 accessions with low-P tolerance and 15 with low-P sensitivity were identified from 560 maize germplasm in field experiments. By analysis of 24 strand-specific RNA libraries from shoots and roots of CCM454 (low-P tolerant) and 31778 (low-P sensitive) that had been subjected to P stress for 2 and 8 days, a general overview of genotypic diversity in maize in response to P stress was provided. The tolerance to low P of CCM454 is mainly due to the rapid responsiveness to P stress and efficient elimination of ROS. These findings increase our understanding of the molecular events involved in the difference in tolerance to P stress among maize genotypes.

## Methods

### Plant growing conditions in field and hydroponic experiments

In 2014 and 2015, 560 maize accessions were evaluated for low-P tolerance in field experiments at Zhangye water-saving agriculture experimental station of Gansu Academy of Agricultural Sciences. The accessions mainly included introgression lines, Chinese elite inbred lines and inbred lines from different ecological zones in China, CIMMYT and the USA. The area (100°26′E, 38°56′N) has a typical arid climate with 150 mm of annual precipitation. The soil at the experimental site was an alkaline (pH 8.5) Orthic Anthrosol and contained 4.72 g/kg Olsen-P. The experiment had an alpha (0, 1) lattice design with two replicate plots for each combination of maize accession and P treatment [32]. The experiment had two levels of P addition, i.e., P was either added or not added. Before sowing, 120 kg P_2_O_5_/ha (or no P_2_O_5_ in the low-P treatment) and 150 kg N/ha were uniformly broadcast and ploughed into the soil. The remaining N fertilizer (150 kg N/ha) was applied by topdressing at the pre-tasselling stage of maize. The following traits were evaluated: plant height, leaf number, normalized difference vegetation index and fresh ear weight. Based on principal component analysis of relative trait values as previously described [40], 15 accessions with low-P tolerance and 15 with low-P sensitivity were identified. Among these accessions, one with low-P sensitivity (inbred line 31778) and one with low-P tolerance (inbred line CCM454) were selected for further research; these two were selected because neighbour joining tree analysis indicated that they are closely related (data not shown).

In a hydroponic experiment, uniform seeds of inbred line 31778 (sensitive to low P) and CCM454 (tolerant to low P) were surface sterilized in 3 % NaOCl for 20 min and then soaked in a saturated CaSO_4_ solution with continuous aeration for 6 h before they were washed three times with distilled water. Seeds were germinated in coarse quartz sand at room temperature until two leaves emerged. After their endosperms were removed, the seedlings were transferred to 3-L pots (three seedlings per pot) supplied with modified half-strength Hoagland’s nutrient solution for 2 days and then supplied with full-strength Hoagland’s nutrient solution containing either 150 μM PO_4_^3−^ (control) or 5 μM (low P) PO_4_^3−^ as indicated. In addition to these two levels of P, the hydroponic solutions contained 0.75 mM K_2_SO_4_, 0.65 mM MgSO_4_ · 7H_2_O, 0.1 mM KCl, 2 mM Ca(NO_3_)_2_ · 4H_2_O, 0.1 mM Fe-EDTA, 1 μM H_3_BO_3_, 1 μM MnSO_4_ · H_2_O, 1 μM ZnSO_4_ · 7H_2_O, 0.5 μM CuSO_4_ · 5H_2_O, and 0.005 μM (NH_4_)_6_Mo_7_O_24_ · 4H_2_O. In the low-P treatments, KCl was added to maintain the same concentration of potassium in both treatments. The maize plants were grown in a growth chamber with 14 h light/10 h dark and a 28/22 °C day/night temperature regime. The nutrient solution was replaced with fresh solution daily to ensure pH stability. Each treatment was replicated three times. As described in the following sections, root and shoot samples were collected at indicated times after initiation of P stress treatment and were subjected to strand-specific RNA-Seq, RNA analysis, elemental analysis, enzymatic assay, and anthocyanin analysis.

### Strand-specific RNA-Seq

Total RNA was extracted from shoots and roots with TRIZOL reagent (Invitrogen, USA), and 3 μg of total RNA was used as input material for RNA library construction. Ribosomal RNAs were removed using Epicentre Ribo-Zero^TM^ Gold Kits (Epicentre, USA). The strand-specific RNA-sequencing libraries were constructed with the NEBNext^®^ Ultra^TM^ RNA Library Prep Kit for Illumina^®^ (NEB, USA). Random hexamers were used for first-strand cDNA synthesis. After second-strand cDNA synthesis, terminal repair and ligation of poly(A)/sequencing oligonucleotide adaptors were carried out. Then, the second-strand cDNA was excised by UNG enzyme. The fragments with expected size were purified and then amplified by PCR. The purified PCR products were sequenced with the Illumina Hiseq 2500 platform (ANOROAD, Beijing, China).

The clean reads were produced after the raw reads were excluded low quantity reads, Ns reads, 5’ and 3’ adaptor contaminants and rRNA sequences obtained from GenBank. Reads that passed the filter were then aligned to the maize B73 RefGen_V3.27 genome. Only perfectly matching sequences were considered for further analysis. The count information was used to determine normalized gene expression levels as RPKM [33]. Multiple testing with the Benjamini-Hochberg procedure for false discovery rate (FDR) was taken into account by using an adjusted *p*-value. Changes in expression were evaluated in response to low P vs. normal P within each line; in response to low P in line 31778 vs. line CCM454; and in response to normal P in line 31778 vs. line CCM454. Genes with statistically significant changes in expression was identified as those with Log_2_ ratio ≥ 1and adjusted *P* value < 0.05 using the DEGseq method [54]. The fold enrichment of various metabolic processes was calculated as described by Chandran et al. [55].

### RNA analysis

The enrichment, fractionation, and detection of miRNA399 from total RNA were performed as previously described [56]. For real-time RT-PCR, first-strand cDNA was synthesized using SuperScript^TM^ III First-Strand Synthesis Supermix (Invitrogen). The cDNA reaction mixture was diluted 20 times, and 1 μl was used as template in a 20-μl PCR reaction. Primers were designed to detect the transcription levels of randomly selected genes. Real-time RT-PCR was carried out in an ABI 7500 system (Applied Biosystems) using the SYBR Premix Ex Taq^TM^ (perfect real time) kit (TaKaRa Biomedicals). Each assay was replicated three times. The comparative Ct method was applied. The primers used in this experiment are listed in Additional file 9.

### Elemental assay

The shoots and roots were heated to 105 °C for 30 min, dried at 65 °C for 72 h and then milled to a fine powder. The weighed samples were then digested in 5 ml of H_2_SO_4_-H_2_O_2_ until the solution became clear. The total P content was determined by the vanadomolybdate method.

### Determination of SOD, POD and CAT activities

About 0.5-g samples of roots or shoots were homogenized in 2.5 ml of 0.05 M phosphate buffer (pH 7.8) and centrifuged at 13,000 × *g* for 15 min at 4 °C. The SOD activity in the clear supernatant was determined according to Constanine and Ries [57]. POD and CAT activities were determined according to Manoranjan [58].

### Root-secretory APase activity

APase activity was determined in the excised roots segments as described previously [59]. After the excised roots was placed in a solution containing 0.5 ml of H_2_O, 0.4 ml of Na-Ac buffer (0.2 mol/L, pH5.2), and 0.1 ml of NPP substrate (0.15 mol/L) for 10 min at room temperature, the reaction was terminated by addition of NaOH. The absorption of the reaction solution was determined at 405 nm.

### Anthocyanin and H_2_O_2_ content

Anthocyanin content in leaves was measured described by Rabino and Mancinelli [60]. Anthocyanin was extracted with 99:1 methanol:HCl (v/v) at 4 °C for 24 h, and the absorbance values at OD_530_ and OD_657_ were recorded. OD_530_-0.25*OD_657_ was used to compensate for the contribution of chlorophyll and its products to the absorption at 530 nm.

H_2_O_2_ content in leaves was determined by measuring the titanium-hydro-peroxide complex as described by Brennan et al. [61].

## Additional files

Additional file 1: Statistics of RNA sequences in roots (A) and shoots (B) in maize inbred lines 31778 and CCM454. (XLSX 14 kb)
Additional file 2: Pearson’s correlation (R value) of biological replicates between roots (A) and shoots (B) in maize inbred lines 31778 and CCM454. (PDF 32221 kb)
Additional file 3: Validation of RNA-Seq by real-time RT-PCR. (A) Up-regulated genes by P stress; (B) Down-regulated genes by P stress. Quantifications were normalized to the expression of *GAPDH*. Values are means and standard errors (*n* = 3). (PDF 189 kb)
Additional file 4: GO classification of common P deficiency-responsive genes between 31778 and CCM454. (PDF 197 kb)
Additional file 5: List of genes with significant expression differences in the shoots of inbred lines 31778 and CCM454 under sufficient condition. (XLSX 417 kb)
Additional file 6: List of genes with significant expression differences in the roots of inbred lines 31778 and CCM454 under P sufficient condition. (XLSX 570 kb)
Additional file 7: List of genes with significant expression differences between inbred lines 31778 and CCM454 after 2 days of P deficiency. (XLSX 153 kb)
Additional file 8: List of genes with significant expression differences between inbred lines 31778 and CCM454 after 8 days of P deficiency. (XLSX 246 kb)
Additional file 9: Oligos used as primers in the experiment. (XLSX 11 kb)

## Abbreviations

APase: Acid phosphatase
CAT: Catalase
DEGs: Differentially expressed genes
DXS: 1-deoxy-D-xylulose 5-phosphate synthase
FDR: False discovery rate
GO: Gene Ontology
IPs: Inositol polyphosphates
MEP: 2-C-methyl-D-erythritol 4-phosphate
P: Phosphorus
POD: Peroxidase
ROS: Reactive oxygen species
RPKM: Reads per kilobase million mapped reads
SOD: Superoxide dismutase
