# Supplementary figures and images for: Strand-specific RNA-Seq transcriptome analysis of genotypes with and without low-phosphorus tolerance provides novel insights into phosphorus-use efficiency in maize
Source: BMC Plant Biol. 2016 Oct 10;16:222. doi: 10.1186/s12870-016-0903-4 (PMC5057381; doi:10.1186/s12870-016-0903-4)

# Supplemental Figure 1

A

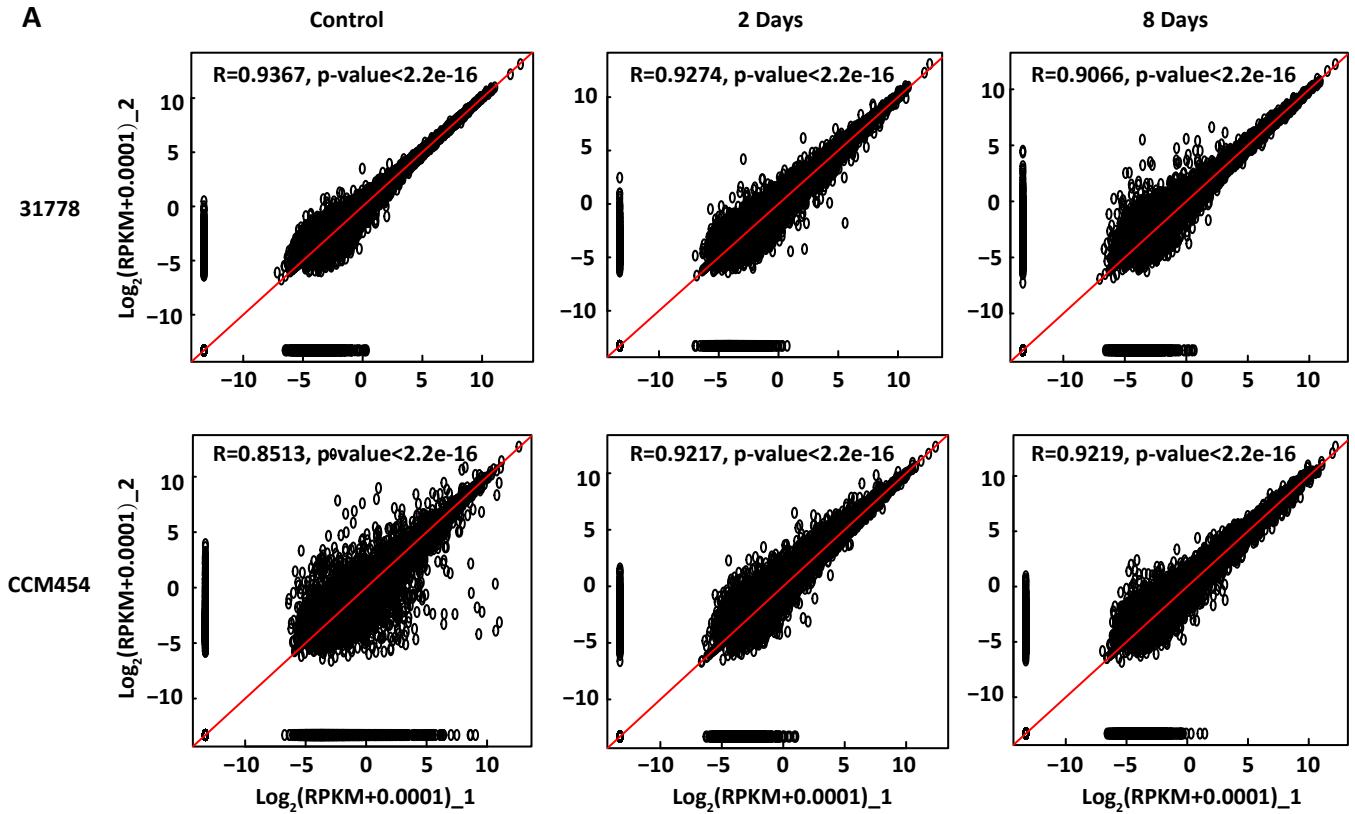

B

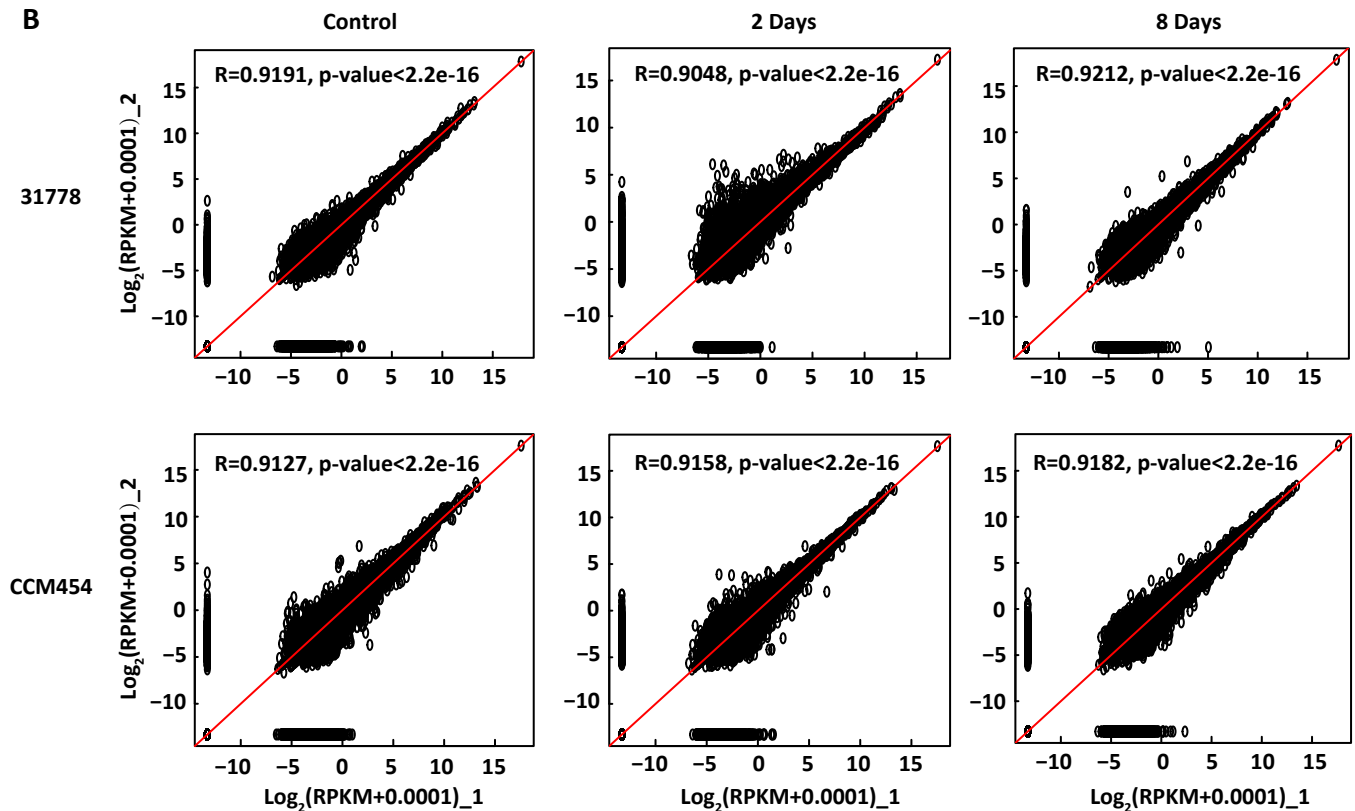

Supplement: Additional file 2: — Pearson’s correlation (R value) of biological replicates between roots (A) and shoots (B) in maize inbred lines 31778 and CCM454. (PDF 32221 kb) [file 12870_2016_903_MOESM2_ESM.pdf]

Supplemental Figure 2

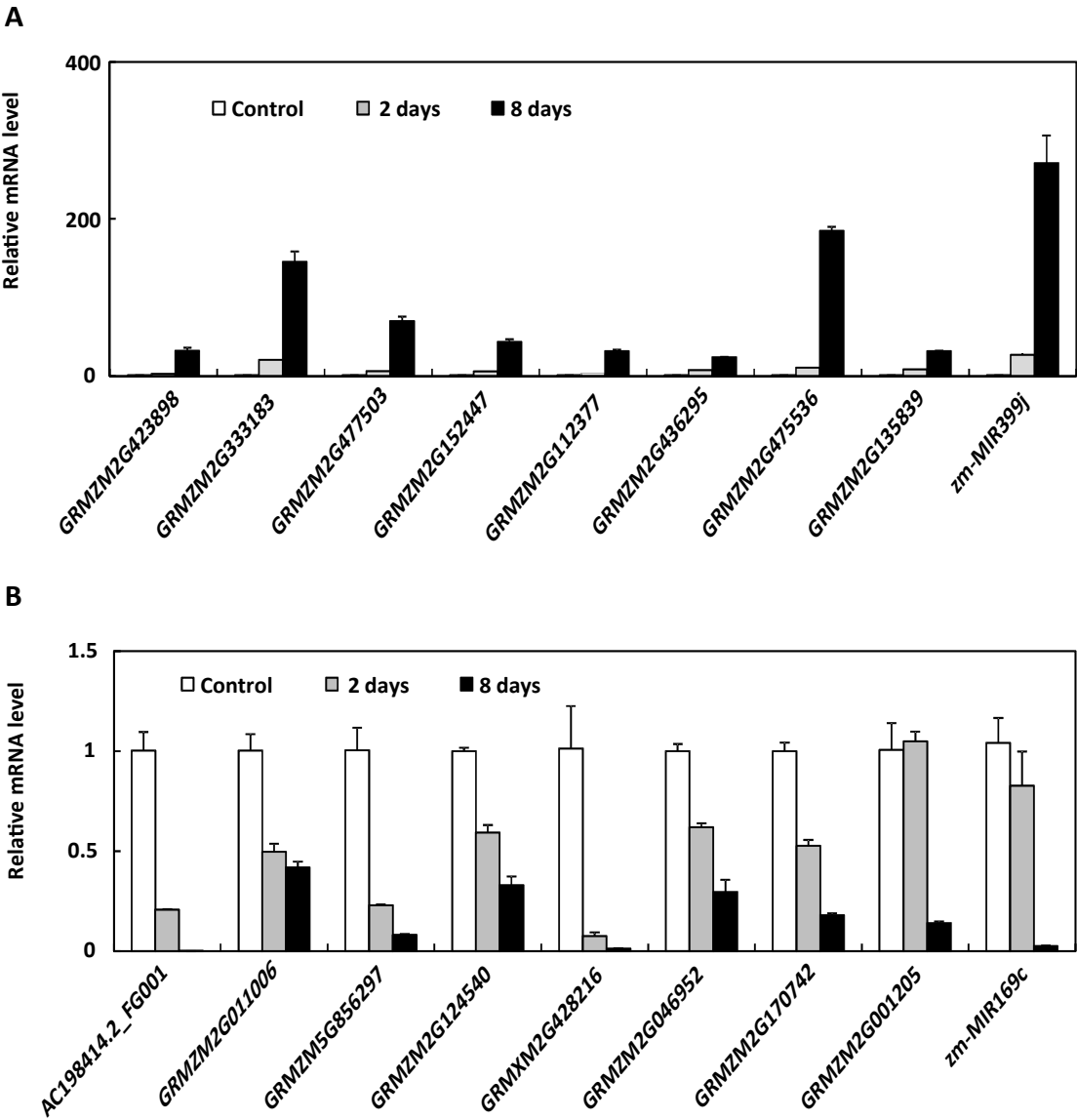

Supplement: Additional file 3: — Validation of RNA-Seq by real-time RT-PCR. (A) Up-regulated genes by P stress; (B) Down-regulated genes by P stress. Quantifications were normalized to the expression of GAPDH. Values are means and standard errors (n = 3). (PDF 189 kb) [file 12870_2016_903_MOESM3_ESM.pdf]

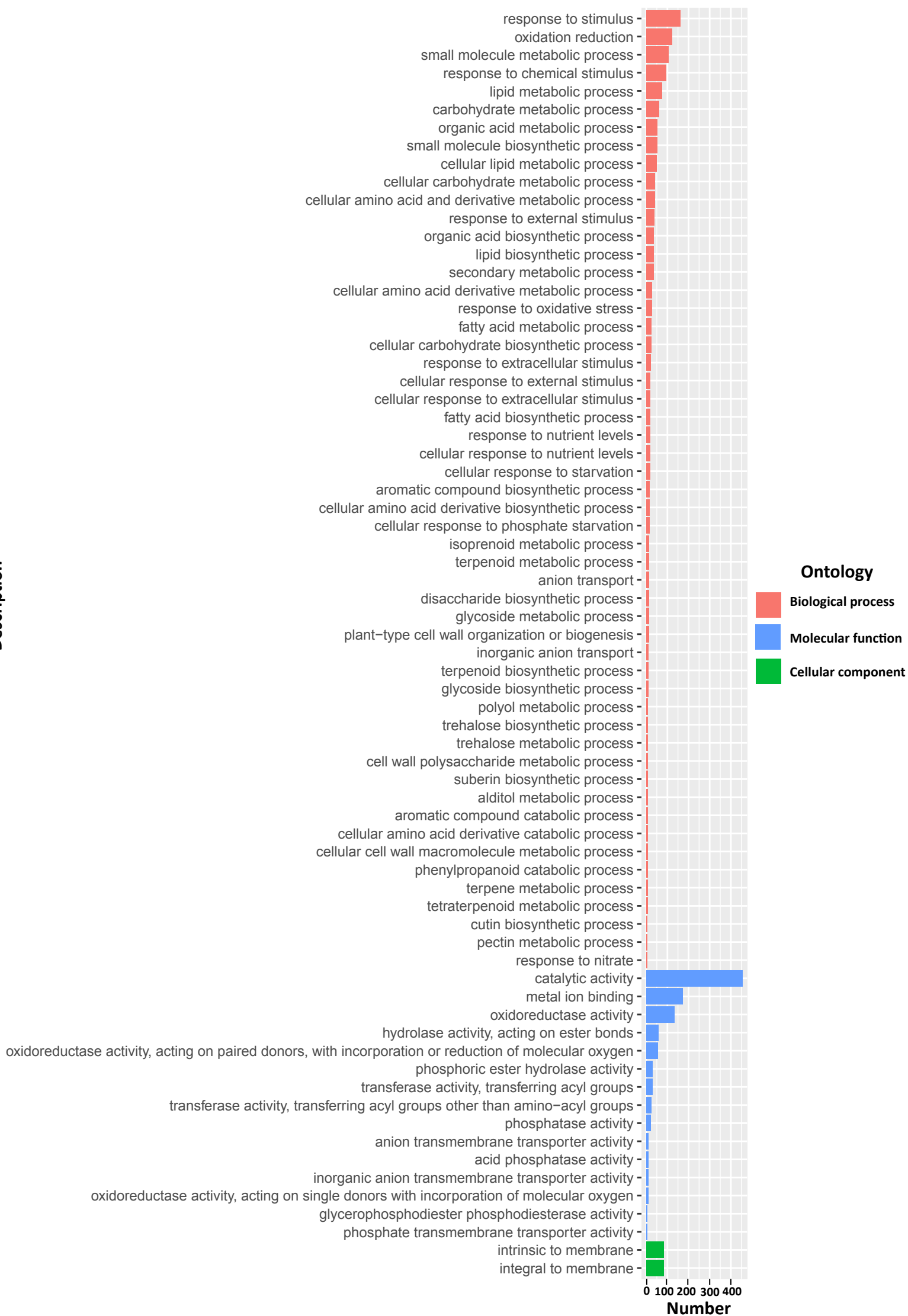

Supplement: Additional file 4: — GO classification of common P deficiency-responsive genes between 31778 and CCM454. (PDF 197 kb) [file 12870_2016_903_MOESM4_ESM.pdf]
